# Trace/Ultratrace Analyses of Unstable Compounds: Investigations on Hydrazobenzene and Azobenzene

**DOI:** 10.6028/jres.093.071

**Published:** 1988-06-01

**Authors:** S. Ahuja, G. Thompson, J. Smith

**Affiliations:** Development Department, Pharmaceuticals Division, CIBA-GEIGY Corporation, Suffern, NY 10901

Trace analysis generally entails determination at parts per million (ppm) or µg/g level. Analyses performed at trace or lower levels (ultratrace) are difficult to carry out for several reasons. The difficulties relate to obtaining a representative sample, avoiding loss or contamination during sample preparation, finding a suitable method for resolving the component of interest without significant loss, and, finally, having sufficient detectability in the range of interest to assure reliable quantitation. These problems are further compounded when one is dealing with compounds such as hydrazobenzene and azobenzene. Discussed below is a method developed to analyze these compounds which circumvents some of the problems encountered with them.

## Experimental

A sample weight anticipated to contain ~ 10 ppm of hydrazobenzene or azobenzene is weighed accurately and shaken with 30 mL of pH 9.2 THAM buffer. This is followed by extraction with 10 mL of n-hexane. After centrifugation, 5 mL of the n-hexane layer is evaporated to dryness, at room temperature, with nitrogen and the residue is solubilized in 1.0 mL of acetonitrile. Twenty-five microliters are immediately injected into HPLC equipped with Partisil 10 µ C_8_ column (25 cm×4.6 mm) and a dual channel detector (254 and 313 nm). Elution is carried out with a mobile phase composed of acetonitrile:acetate buffer, pH 4.1 (11:14). Both hydrazobenzene and azobenzene standards are treated similarly.

## Results and Discussion

**Figure 1:**
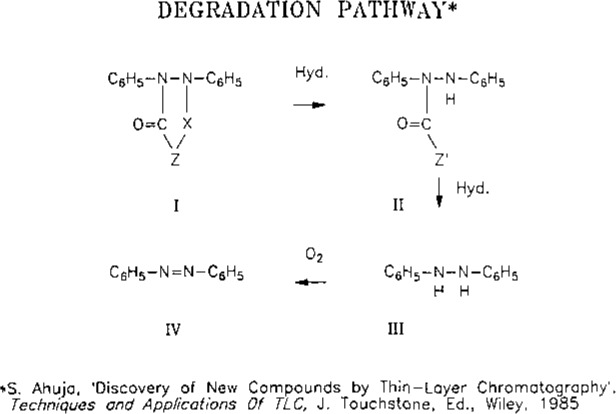
Degradation pathway of parent compound.

A review of the literature revealed that a normal phase HPLC method has been reported for the analysis of hydrazobenzene and azobenzene [1]. The method entails extraction of these compounds into n-hexane from 1*N* NaOH followed by analysis on Partisil-10 PAC column with a mobile phase containing 2.5% absolute ethanol. The published method suffers from the following shortcomings:

- Hydrazobenzene and azobenzene show significant instability in 1*N* NaOH (table 1).
- Azobenzene can isomerize into cis- and trans-isomers. Their separation is not demonstrated or accounted for in the method.
- Parent compound (I) can degrade directly or indirectly into hydrazobenzene and azobenzene (fig. 1).
- Selectivity of transformation products given in figure 1 is not demonstrated.

**Figure 2:**
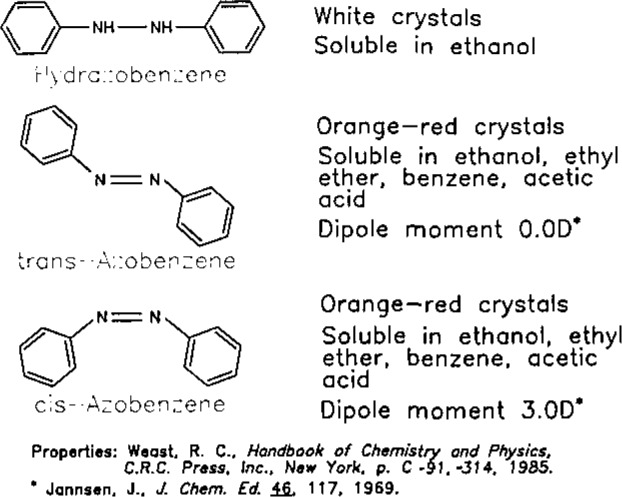
Physical properties of hydrazobenzene and azobenzene.

The properties of hydrazobenzene and azobenzene are given in figure 2. Hydrazobenzene is known to be an unstable compound, it oxidizes easily to azobenzene and other compounds and has *t*_1/2_ of 15 minutes in wastewater [2]. Azobenzene, on the other hand, can isomerize or sublime even at 30 °C [3].

To assure that the methodology would be reliable at ~10 ppm, suitable methods were developed for detecting these compounds at levels ⩽ 1 ppm, i.e., ultratrace levels. To further assure reliability of analyses, an effort was made to meet the following requirements for ultratrace analysis [4]:

- Sample used for analysis was representative of the whole lot.
- Methodology incorporated optimum separation and detection techniques.
- Component of interest was allowed to suffer a minimum loss during various analytical operations.
- Adequate steps were incorporated in the analytical method to account for losses that might occur due to sample preparation or degradation.

Furthermore, to assist other researchers in evaluating whether these methods could be useful for their investigations, the following analytical parameters were included:

Amount Present in Original Sample (APIOS)
Minimum Amount Detected in g (MAD)
Minimum Amount Quantitated in g (MAQ)

**Figure 3:**
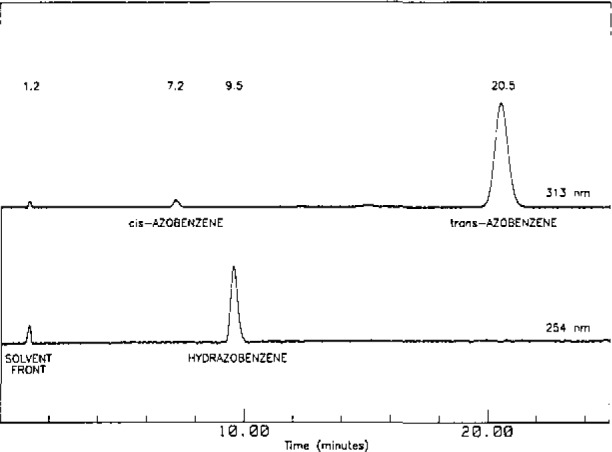
Chromatograms of azobenzene (cis- and trans-) and hydrazobenzene (0.05 µg of each compound injected and monitored at 313 nm and 254 nm).

Investigations revealed that the optimum pH for extraction for both hydrazobenzene and azobenzene is 9.2. At this pH, these compounds can he easily extracted from the parent compound and are quite stable (table 1). The cis- and trans-isomers of azobenzene and hydrazobenzene can be resolved well with the reversed-phase HPLC method (fig. 3). Previous investigations had confirmed the selectivity of this method as it resolves compound I (*t*_R_≃11 minutes) from other transformation products [5]. Data on spiked samples are given in table 2. An average recovery of 91% and 114% was obtained for hydrazobenzene and azobenzene respectively with relative standard deviation (R.S.D.) of 3–11%. The methods were found useful for quantitating ⩽ 1 µg of these compounds with respect to the parent compound (MAD = 6–7 ng). The high recovery obtained for azobenzene is partly due to conversion of hydrazobenzene to azobenzene (~9%). Further improvements are being investigated.

## Conclusions

1. Selective methods have been developed for analysis of hydrazobenzene and azobenzene.
2. The instability of hydrazobenzene in aqueous and organic solvents is well known. This problem has been effectively dealt with in that an average recovery of 91% was obtained for the active ingredient, capsules and tablets.
3. It was found that azobenzene is susceptible to isomerization and sublimation. The developed procedure provides an average recovery of 114% for the active ingredient, capsules and tablets. The high values are partly due to conversion of hydrazobenzene to azobenzene (~9%).
4. The developed methods provide reliable values (3–11% R.S.D.) for hydrazobenzene and azobenzene at a concentration of ⩽10 µg/g (⩽10 ppm) in terms of the parent compound.

## Figures and Tables

**Table 1:** Stability of hydrazobenzene and azobenzene

Medium	Time	%Loss	
		Hydrazobenzene	Azobenzene
0.1*N* NaOH	30 minutes	82.9%^a^	4.6%^b^
pH 9.2 buffer	30 minutes	0.9%^c^	None found^d^

Original concentration in 10% acetonitrile:

a 11.8 µg hydrazobenzene/mL.

b 15.7 µg azobenzene/mL.

c 3.55 µg hydrazobenzene/mL.

d 2.59 µg azobenzene/mL.

**Table 2:** Recovery data of spiked samples. APIOS: ⩽ 10 µg/g of parent compound

Sample	Hydrazobenzene found	Azobenzene found
Parent compound	89.0±8.6% (n = 7)	123±2.6% (n = 6)
Capsules	89.6±10.8% (n = 5)	98.7±10.2% (n = 3)
Tablets	95.8±5.4% (n = 3)	121±4.2% (n = 3)
Average	91%	114%
R.S.D.	±5–11%	±3–10%
MAD (µg)	0.006 (6 ng)	0.007 (7 ng)
MAQ (µg/g)	⩽1	⩽1

## References

[1] Matsui F, Lovering EG, Curran NM, Watson JR (1983). J Pharm Sci.

[2] Riggin RM, Howard CC (1979). Anal Chem.

[3] Weast RC (1985). Handbook of Chemistry and Physics.

[4] Ahuja S (1986). Ultratrace Analysis of Pharmaceuticals and Other Compounds of Interest.

[b5-jresv93n3p344_a1b] 5Ahuja, S., Shiromani, S., Thompson, G., and Smith, J., Personal Communication, March 2, 1984.

